# The role of the physician in Israel’s maternal child health clinics: surveys of professional and parental perceptions

**DOI:** 10.1186/s13584-017-0174-z

**Published:** 2017-10-02

**Authors:** Chen Stein-Zamir, Hannah Shoob, Deena R. Zimmerman

**Affiliations:** 1 Jerusalem District Health Office, Ministry of Health, 86 Jaffa Road, 94341 Jerusalem, Israel; 2 Braun School of Public Health and Community Medicine, Faculty of medicine, the Hebrew University of Jerusalem, Ein Kerem. PO Box 12272, 91120 Jerusalem, Israel

## Abstract

**Background:**

Preventative health services are a pediatric health care cornerstone, which strives to promote health and prevent illness and injury. In Israel, Maternal Child Health Clinics (MCHC) provide these well child services for ages 0–6 years. MCHC care includes physician visits; however, the physician’s role is not well defined. The study purpose was to provide a basis for setting policies that determine the role of physicians in the provision of MCHC services. To get broad input we included MCHC stakeholders - parents, MCHC physicians, non-MCHC physicians and MCHC nurses, specifically to obtain insights regarding the MCHC physician role and to characterize the stakeholder demographics, service utilization, and practice patterns.

**Methods:**

Professional groups completed self-administered written questionnaires (*n* = 398). Parents were interviewed during MCHC visits using a structured questionnaire (*n* = 1052). All provided demographic data, service characteristics and agreement with ten potential MCHC physician roles - Physical Examination, Abnormal Health Condition Detection, Developmental Screening, Anticipatory Guidance, Parent-Child Interaction Counseling, MCHC Staff Advice, Children-at-Risk Detection, Growth Surveillance, Vaccination Counseling, and Inter-physician Communication.

**Results:**

The study findings seem to indicate a true shortage of MCHC physicians. The median age of MCHC physicians was significantly higher than both non-MCHC physicians and MCHC nurses. There was agreement among stakeholders regarding some roles (Physical Examination, Developmental Screening and Detection of Abnormal Health Conditions) but not others. Most parents reported having at least one MCHC physician encounter. Parents who did not visit the physician were younger and had fewer children.

**Conclusions:**

Stakeholders view MCHC physicians as integral to MCHC care. Roles traditionally regarded as part of primary prevention were less likely to be attributed to physicians than screening roles considered secondary prevention.

Updating and standardization of the MCHC physician role is needed along with a national strategy to recruit and train MCHC physicians.to ensure optimal pediatric preventive health care in Israel.

## Background

Preventative health services are one of the cornerstones of pediatric health care [1]. While there is universal consensus that the main goals of well child care are primary prevention of illness and injury and early detection of medical conditions, there is wide variation worldwide in the organization and delivery of such care [2–4]. Israel’s pediatric preventative health services are provided by community-based Maternal Child Health Clinics (MCHC), which are operated separately from curative health care.

The first MCHC (known locally as “Tipat Chalav” - drop of milk), was established in Jerusalem one hundred years ago. MCHC care was associated with a marked reduction in infant morbidity and mortality [5]. There is high acceptance of this free universal community-based service by the population, with an estimated overall use of over 95% [6].

Israel’s Ministry of Health (MOH), through its regional District Health Offices, runs the MCHC that provide care for two thirds of Israel’s children; the municipalities of Jerusalem and Tel Aviv care for 14%; and the health funds provide care for the remaining 20% of children [7]. Multiple professional committees have been convened to examine MCHC service provision [8], including one that is currently ongoing.

MCHC preventative health care by all providers is given according to a standardized model guided by MOH directives, with government-funded routine childhood immunizations. The services offered include immunizations, physical health monitoring, neuro-developmental surveillance and guidance for families regarding child development, nutrition, behavior and common health conditions. Since the inception of the MCHC, the standard model has included care provided by a team of public health nurses and physicians. The role of the public health nurse during each visit has been laid out in great detail [9].

According to the most recent MOH guidelines (2004), routine nurse MCHC visits are scheduled at ages 2 weeks; 1, 2, 4, 6, 9, 12, and 18 months; and 2.5, 3.5 and 5.5 years. Routine physician visits are scheduled at ages 2, 9, and 18 months; and 2.5 and 5.5 years. Additional nurse and/or physician visits can be scheduled if clinically needed. Services at all visits include basic measurements such as height and weight, developmental evaluation, and parental guidance. Head circumference is measured through 18 months of age [10]. Vaccinations are administered at ages 1, 2,4,6,12,18 and 24 months [11]. At nine months, the child is referred for complete blood count screening for anemia; and the last two visits include vision screening.

In the MOH guidelines, the role of the MCHC physician is defined as follows:

Every physician visit will include history taking, physical examination and neuro-developmental evaluation to determine the child's developmental status.

At the time of the study, these components were not defined; the difficulty in providing consistent service with undefined roles was the main impetus for this study.

Studies of overall MCHC services have shown variation in parental attitudes towards different components of the MCHC service. In their study of 963 mothers, Palti et al. reported that, while most mothers brought their children to the MCHC nurses’ care, almost 10% had never brought their child for MCHC physician evaluation [12]. In a 2007 study, Rosen et al. reported that parental satisfaction with nurses’ professionalism and patient relations was 66% and 73% respectively, while satisfaction for physicians was 46% and 48% respectively [13].

The purpose of this study was to provide a basis for setting policies that determine the role of physicians in the provision of MCHC services. In order to get broad input, we included families who receive MCHC care and professionals - physicians and nurses who provide MCHC care, and non-MCHC pediatricians. Specific aims were a) characterize the stakeholder demographics and service utilization and practice patterns and b) obtain insights of the four stakeholder groups regarding the role of the MCHC physician.

## Methods

### Study plan

The study was conceived as a cross sectional survey among four groups of stakeholders:

1. MCHC Physicians – Physicians who provide services according to the national program of pediatric preventative health care in government or health fund run MCHC. These physicians can work either full-time in a MCHC or have part-time employment in a MCHC in conjunction with primary care responsibilities in a health fund curative clinic.
2. Non –MCHC Physicians – Practicing pediatricians who work in hospital-based or primary care settings (or both) but do not work in any MCHC.
3. MCHC Nurses - Public Health nurses who underwent specific professional training in pediatric preventative health care and currently work in a MCHC, run by the government (national or municipal) or a health fund.
4. Parents – Mothers or fathers who have at least one child being cared for in a MCHC during the study period.

### Study instrument

For each group of participants, we designed a specific questionnaire. The questionnaires included a list of ten potential roles for the MCHC physician - Physical Examination, Abnormal Health Condition Detection, Developmental Screening, Anticipatory Guidance, Parent-Child Interaction Counseling, MCHC Staff Advice, Children-at-Risk Detection, Growth Surveillance, Vaccination Counseling, and Inter-physician Communication. This list was based on 1) review of the international literature on well child care; 2) national and international guidelines for pediatric preventative health care; 3) Delphi process with experts in the field of preventive health care for children in Israel; and 4) the researchers’ professional experience in the provision, supervision and auditing of MCHC care. Study participants were asked regarding the degree of agreement for each of the ten roles on a four point Likert scale (Strongly agree, agree, disagree, strongly disagree) and were also asked to list the top three roles.

The first section of all questionnaires included demographic variables (age, gender, country of origin). For professional groups, data were obtained as to location of medical or nursing school they attended and employment setting. Parents were asked the number of children in their family and the age of the youngest child. Each questionnaire also included items related to MCHC services adapted to the particular group of stakeholders. Physicians were queried regarding interaction with their colleagues. This included the referral frequency for a list of ten common situations (anemia, developmental delay, growth deviance, emotional difficulties, nutrition, orthopedic conditions, speech pathology, medical conditions and prematurity-related issues). They were also asked their opinion on the optimal number of physician visits and timing of the first visit. Parents were questioned about their expectations regarding MCHC care and experiences. They also were questioned about service utilization by the child whose MCHC visit was occurring at the time of the interview. Parents were asked if they had been given a referral for further evaluation in their health fund for any of their children and if they followed through with the referral. Each questionnaire was pre-tested on a small group of participants and revised accordingly.

### Questionnaire administration

#### Professional groups

We estimated that there should be 150 MCHC physicians nationally (*n* = 150). This number was derived by dividing the annual number of live births (178,723 in 2015) by 1250, the number of infants assigned to each MCHC physician full time position by Ministry of Health Directives [9].

We tried to recruit 150 non-MCHC pediatricians and 150 MCHC nurses. These professionals were recruited to complete a self-administered questionnaire in a number of ways. Since the Ministry of Health medical and specialty license registry does not include information regarding type or location of current practice [14], we attempted to reach a varied group of pediatricians via national meetings. The questionnaires for both physician groups were distributed during two bi-annual meetings of the Israel Ambulatory Pediatric Association (the organization of community-based pediatricians) and the annual meeting of the Israel Clinical Pediatric Association (the organization of hospital-based pediatricians) between February 2015 and February 2016. These are the annual national meetings with the highest attendance of a broad spectrum of pediatricians. Physicians working in both primary care and MCHC received an MCHC physicians’ questionnaire. The desired number of 150 non-MCHC physicians was achieved in this manner as well as information from some MCHC physicians. Further recruitment of MCHC physicians was done at a session of a monthly continuing medical education meeting of MCHC physicians. All attendees of one meeting completed the questionnaire; additional outreach was made to locate MCHC physicians by personal contact and email. Despite these extensive efforts, the final number of MCHC physicians obtained was 97, probably reflecting that the actual number of MCHC physicians falls short of the calculated estimate.

The nurses’ questionnaires were distributed at the November 2015 meeting of the Israel Pediatric Association (the national union of pediatricians), which included a session for MCHC nurses employed by all providers. Questionnaires were also distributed via the head nurses of district offices. Recruiting 150 MCHC nurses was achieved.

#### Parents

The sample size of parents was determined by assuming that the distribution of answers as to the MCHC physicians’ roles cannot be predicted in advance. Therefore, grouping the distribution of parental replies into 2 main groups – 2 degrees of “agree” versus “disagree” led to a 50/50 probability. Assuming a margin of error of 5% and a confidence interval of 95%, the sample size calculation resulted in 1061 parents.

All parents were administered a structured interview by one trained research coordinator between January and August 2016. These face-to-face interviews took place during routine MCHC visits. MCHC were selected via stratification based on geographic region in Israel and population characteristics such as ethnicity (Arab/ Jewish) and religious affiliation (Jewish Ultra-Orthodox/Traditional/Secular). The parental refusal rate was minimal (9/1061, primarily due to lack of time).

### Statistical analysis

The data were coded and entered into Microsoft Excel worksheets. Data analysis was performed with SPSS® (SSPS Statistics for Windows, Version 22) and WINPEPI® (PEPI for Windows, Version 11.65) software. The characteristics of the study populations were described and analysed as follows: Continuous variables were compared by Student t test; dichotomous variables were analyzed by Pearson chi-square test; medians were compared using the Independent Samples Median Test and comparisons of proportions were performed by calculating Odds Ratio (OR) with 95% confidence intervals (95%CI), in all analyses. A logistic regression model was performed for the dependent variable – MCHC physician visits – including the independent variables child age, gender, ethnicity, parental country of birth and number of children in the family.

Perceptions regarding the 10 roles of the MCHC physician were analyzed by several methods. The responses of the four study groups of study participants as to the degree of agreement (scale 1–4) were mapped. Then, a multiple-comparisons procedure (Tukey’s procedure) was performed on the proportion of participants replying, “strongly agree” with each role. Based on the listing of 3 top ranked roles in each group, a cumulative score was assigned to each physician role.

A *p* value <0.05 was considered significant in all comparisons.

### Ethics approval

This study was approved by the Institutional Review Board of the Israel Ministry of Health, approval number 144–2014.

## Results

### Study population characteristics

#### Professionals

Written Questionnaires were completed by 398 professionals (Table 1).

**Table 1:** Professional Group Characteristics

	MCHC Physicians *N* = 97	Non - MCHC PhysiciansN = 151	MCHC Nurses *N* = 150
Female Gender	63 (65.6%)	70 (46.7%)	100%
Average Age	56.3 ± 9.7	49.6 ± 12.7	48.3 ± 10.7
Median Age	59	50	49
Country of Origin			
Israel	29 (29.9%)	105 (70%)	83 (55.3%)
Former Soviet Union	52 (53.6%)	14 (9.3%)	23 (15.3%)
Other	16 (16.5%)	31 (20.7%)	44 (29.5%)
Years of Work Experience (mean ± SD)	28 ± 10.2	20.9 ± 13	22 ± 10.8
Years of Work Experience (median)	30	21.5	23

In almost all cases, the country of professional education was the country of origin. The main place of work was reported for 135/151 non-MCHC physicians; most (87%) were community-based and 13% were hospital-based. Of the community-based physicians, 56%, 21%, 20% and 3% worked for “Clalit”, “Maccabi”, “Meuhedet” and “Leumit” health funds, respectively.

#### Parents

The final parental group consisted of 1052 participants interviewed during MCHC visits. Demographic data for the parents are presented in Table 2.

**Table 2:** Parental Demographics

Age (mean ± SD) years	30.8 ± 5.4
Age (median)	31
Male gender	104 (9.9%)
Jewish	857 (81.5%)
Arab	195 (18.5%)
Highest Level of Education	
High School	305 (29.0%)
Teacher’s Seminary	88 (8.4%)
Bachelor’s Degree	383 (36.4%)
Master’s Degree	162 (15.4%)
Other	114 (10.8%)
Number of children (mean ± SD)	2.8 ± 1.98
Number of children (median)	2
Number of children (range)	1–16

The ethnic distribution generally reflects that of Israeli population. The large range of number of children per family reflects the diversity within Israel’s sub-populations.

#### Expectations

Parents were asked an open-ended question regarding their expectations from MCHC physician visits. The two main expectations mentioned were to assure the parents that “the child is ok” - without medical issues (52.2%), and developmental monitoring (43.1%). Few parents (3.8%) felt that the visit is unnecessary (i.e. duplicated appraisal by the health fund pediatrician) and less than 1% could not offer a purpose.

#### Service utilization

Most parents (*n* = 878, 83.5%) reported having visited the MCHC physician at least once with the child who was the focus of the interview. Most of those who had not visited the physician reported that either they had not been given an appointment, or that the child was already seen by a non-MCHC physician.

Of the 878 children who visited the physician, 142 children were examined at an earlier age than the recommended first MCHC physician visit at age 2 months. This reflects the prerogative to schedule visits if felt to be indicated by the health professionals. Many infants (*n* = 124) were seen at exactly 2 months of age, a time at which a number of routine vaccinations are recommended. The remainder were seen above the age of 2 months.

Service utilization patterns of children aged over 2 months were investigated (*n* = 684). At least one MCHC physician visit was reported in 612 (89.5%) children. The parents who did not visit the MCHC physician were compared to the parents who visited. Parents who did not visit the MCHC physician were younger (mean 29 ± 5.9, median 28 vs. 31.1 ± 5.3, median 31, *p* < .002) and had fewer children (mean 1.9 ± 1.5, median 1, 2.9 ± 2, median 2, *p* < .0001) compared to parents who brought their child to the visit. A logistic regression analysis model was implemented to evaluate characteristics associated with bringing the child to the MCHC physician visit at least once. The variables included: child’s age, gender, ethnicity, parental country of origin and level of education (of the parent interviewed) and the number of children in the family. Parents with a single child were less likely to report a visit, compared to parents with two or more children (77.8% vs. 94.4%, Odds Ratio 5.2, Confidence Interval 3.1–8.8, *p* < .0001). None of the other variables was found to be significantly associated.

#### Physician roles and services

**Fig. 1:**
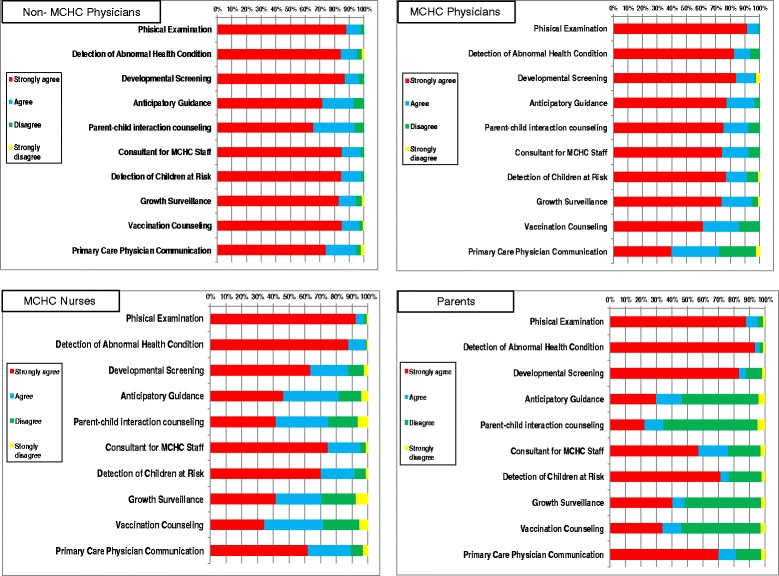
Degree of Agreement with Potential MCHC Physician Roles by Group

The distribution of the degree of agreement with each of the 10 potential MCHC physician roles, among the 4 study groups is presented in Fig. 1. There are areas of agreement and disagreement in perceptions of the roles between the four groups. Comparison of the 4 groups regarding the proportion of responders who listed “Strongly Agree” to each of the 10 MCHC physician roles was carried out using multiple pairwise comparisons (Tukey Procedure). The distribution is presented in Table 3 (with the items that are statistically significant indicated in bold).

**Table 3:** Distribution of Respondents who Replied “Strongly Agree” with specific MCHC physician roles in the 4 Study Groups

	MCHC Physicians		Non-MCHC Physicians		Nurses		Parents	
	N	%	N	%	N	%	N	%
Physical Examination	86/94	91.5	129/146	88.4	137/148	92.6	923/1050	87.9
Detection of Abnormal Health Conditions	77/93	82.8	124/147	84.4	130/148	87.8	981/1049	*93.5* *
Developmental Screening	79/94	84	126/145	86.9	94/148	*63.5* **	876/1050	83.4
Anticipatory Guidance	73/94	77.7	104/145	71.7	68/146	46.6**	312/1050	29.7* *
Parent-child interaction counseling	70/93	75.3	95/145	65.5	60/144	*41.7* **	235/1050	22.4**
Consultant for MCHC Staff	70/94	74.5	125/147	85	110/147	74.8	602/1048	*57.4* **
Detection of Children at Risk	71/92	77.2	121/143	*84.6* *	104/148	70.3	749/1050	71.3
Growth Surveillance	69/93	74.2	121/146	82.9	61/146	*41.8* **	426/1050	*40.6* **
Vaccination Counseling	57/93	61.3	123/145	84.8	50/145	34.5* *	358/1050	*34.1* **
Primary Care Physician Communication	36/90	*40* **	108/146	74	92/148	62.2	737/1050	70.2

*p < 0.01

**p <0.001

In both Fig. 1 and Table 3, MCHC physicians had a more limited view of their role than non-MCHC physicians did. Nurses perceived the role of the MCHC physician differently than both groups of physicians. The parents’ group perceptions were more similar to those of the nurses than to those of the physicians.

The three top ranked MCHC physician roles, agreed upon by all groups, were developmental evaluation, physical examination and abnormal health condition detection. Regarding least important roles, there was less agreement. MCHC physicians and nurses listed growth monitoring and vaccine counseling low. All groups except MCHC physicians ranked providing anticipatory guidance as low.

The lowest degree of agreement by the MCHC physicians was with their role in communication with the curative health care system. MCHC physicians who originated from the former Soviet Union were significantly less likely (26% of the former Soviet Union vs 54% from those with other origins, *p* = 0.005) to agree with this role. No other demographic variables of the physicians had any association with the degree of agreement regarding physician roles.

The recommended age of the first visit was two months (median) in all groups except non-MCHC physicians who recommended an earlier visit (median 1 month). All groups recommended adding an additional physician visit in the first year of life for a total of three instead of the currently scheduled two visits.

The most common reasons for referral to the health fund physician reported by MCHC physicians were suspected hearing problems, orthopedic problems and anemia. The least referred problem was nutrition. Over one third of parents (*n* = 414), reported that they had been given a referral for any of their children. Considering the total number of parents (*n* = 1052) and average number of children per family (2.8), the estimated overall mean referral rate is about 14%. Most (97%) parents reported complying with the referral.

Regarding contact of the MCHC physician with the referred child’s primary care physician, 9.5% replied “always”, 23.8% “frequently”, 63.5% replied “rarely” and 3.5% “never”. Regarding receiving any response (written or oral) from the child’s primary care physician, the replies were: 13.5% “frequently”, 45.8% “rarely” and 40.7% “never”. When non-MCHC physicians were asked what they did upon receiving a referral, responses varied by medical conditions. For some conditions, e.g., nutrition, orthopedic problems, speech delay and hearing, they were more likely to follow the request and generally to give a further referral for specialist or paraprofessional care. For other conditions such as pre-existing medical illnesses, deviance from expected growth and development, they were more likely to re-evaluate the child and then decide what to do next.

As to communication within the MCHC professional teams, the majority of nurses stated that they work in partnership with the MCHC physician all or most of the time (72%) and that they are informed of all out-of-MCHC referrals (85%). The majority of nurses view follow-up of physician referrals as their role either solely (76%) or in conjunction with the physician (24%).

## Discussion

Our findings reflect a number of major challenges regarding MCHC physicians in Israel. As noted, we only found 97 such physicians. This is lower than expected and does not provide enough of a workforce to meet the mandated 5 physician well child care visits. Furthermore, the age distribution indicates that within the coming decade most of the current MCHC physicians will retire. The national fraction of physicians over the age of 45 years is about 2/3 [14, 15], similar to non-MCHC physicians in our study. For the MCHC physicians, the percentage was considerably higher (84%). The need is acute as MCHC physician services continue to be highly accessed by the Israeli public. A further concern, however, is that the younger and first-time parents have been less likely to adhere. This suggests that there is a need for targeted outreach to the younger generation of parents which would increase the need for MCHC physician services.

Another challenge is the lack of uniformity in understanding the role of the MCHC physician. All groups ranked performance of a physical examination and detection of abnormal health conditions among the top three MCHC physician roles. This finding meets parental expectations that the purpose of MCHC physicians is to "make sure that the child is OK." These roles represent mainly secondary prevention (screening) capacities. Activities traditionally considered primary prevention such as growth monitoring and vaccination counseling were less likely to be perceived by as MCHC physician roles. The parents in our study do not see counseling and guidance as a core component of the physician’s role. This differs from findings in other countries where well child care is provided by physicians [16–18]. This may be a reflection of the current hospital-based training of Israel’s pediatricians and their lack of exposure to developmental and behavioral pediatrics [19].

Another major challenge in MCHC care delivery is communication between the preventive and curative health services. Good continuity of care is an important component of medicine that has been shown to improve health outcomes [20]. The lack of continuity of care between the MCHC and health fund primary care physicians was previously noted by.Rosen et al. where only 10% of parents were satisfied with situation [13]. Our findings provide the opportunity for more in-depth understanding of areas of strength and weakness in communication. First, they suggest good teamwork between MCHC physicians and nurses. Second, parents respect referrals given by MCHC physicians and follow through by bringing these referrals to the curative health system. For many situations, the non-MCHC physician respects the recommendation of the MCHC physician. On the other hand, there is almost no direct communication between the two groups. By protocol, all MCHC MD referrals are done through the electronic health record and generally include pertinent information such as the growth chart. However, the MCHC team only hears the outcome of the referral when reported by the parents at the next visit. This one-way written communication suggests a need for improving collegial relationships between those mutually entrusted in the medical care of children.

Previous research, among parents only, revealed mixed results with regard to parents’ satisfaction with MCHC care [12, 13]. These studies, however, did not include inquiries about the physician’s role. The results of our study suggest that parents do perceive the physician as an important part of MCHC care. Further research is needed to explore Israeli parents’ satisfaction with their primary care physician (non-MCHC) in the health fund clinics and to investigate if the findings to date are a reflection of MCHC care or pediatric community care in Israel in general.

### Strengths and limitations.

This study represents a comprehensive attempt to determine the opinion of professionals regarding Israel’s preventive health services. Based on 2012 Ministry of Health data as to the number of pediatricians working in Israel, our study surveyed the opinions of over a fifth of them [14]. We also obtained information from a large cohort of parents. This updates the last study of parents’ perception of MCHC care which was conducted almost a decade ago.

The main limitation of the study is the representativeness of the participants due to non-random sampling. For physicians, this is due to the lack of detailed data regarding place of employment for Israel’s physicians. However, we believe our efforts in finding a large sample via diverse meetings assured us reasonable representativeness. For parents, we sampled based on demographic characteristics of the MCHC clinics they attended. We were able to determine some components of ethnic origin (Jewish vs.Arab, and within Jewish, Ultraorthodox) since we did not question parents regarding their degree of religiosity or income due the sensitive nature of these topics. Due to the terms and conditions of the MOH Ethics Approval Committee, we were able to approach only parents who did use the services of the MCHC. However, the lack of interviews with parents who did not use MCHC services should not impact on the findings of the study as they would have no experience on which to draw their opinions.

### Policy implications

It is important to have physicians at MCHC sites to address the impact of illnesses, acute and chronic, minor and major, on growth and development. An MCHC physician is in the ideal position to integrate the information obtained during the public health nurses’ structured screening to determine the need and urgency of referral for further evaluation.. The physician is also in a position, when appropriate, to reassure parents and minimize unnecessary parental anxiety. This is part of MCHC physician training and continuing medical education. Having an onsite MCHC physician who communicates with his professional colleagues can regulate referrals to the already over-crowded curative health care system [21]. This communication can be greatly facilitated by assuring electronic communication between the curative and preventive electronic health care records. This has begun in the area of immunization records [22] but must be expanded.

The process of structural redesign of the MCHC physician role should be purposeful and evidence based. The components to be addressed should include number and timing of physician visits and target population. The redesign should assure adequate time to meet parents needs [23]. Alongside all improvements and adjustments to the MCHC system, there must be ongoing outreach to parents, especially first time parents, regarding the importance of preventive health care services for children. This should include the importance of team based MCHC care that includes the important contributions that can be made by the MCHC physician.

The number and demographics of the MCHC physicians indicate that there is a critical need for organized efforts to recruit community-based preventative care physicians. These efforts should include declaring pediatric preventive care a medical specialty in crisis [24] and providing financial and professional incentives to entice physicians to provide this service. This process should involve redesign of employment structure, updating the physician role, and providing academic activities. Structural redesign should include facilitation of combining of MCHC work and acute care. Health care organizations should cooperate with workforce sharing. Striving to eliminate bureaucratic barriers (e.g. tax disincentives for having multiple employers, unrealistic minimal hours of work) for the individual physician. This combined position may help minimize the burn out currently experienced by many Israeli primary care physicians and improve quality of care [25]. Clear MOH directives should specify the roles of the MCHC physician that include providing more than just physical examination. First steps have been taken in this direction with the composition of guidelines for each well-child visit, standardization of training for new physicians and an ongoing continuing medical education program for all MCHC physicians. Academic advancement via community-based teaching and research may also empower the MCHC physician.

## Conclusions

The MCHC physician is viewed by parents and colleagues as integral to the preventive care of children. The MCHC physician service is utilized by most parents surveyed, although probably less than the currently mandated visits. This study indicates that the there is need for ongoing update and standardization of the professional role of the MCHC physician, as well as a need for an organized national framework is needed to recruit and train the future generation of MCHC physicians.
